# Supplementary material for: Plant roots increase both decomposition and stable organic matter formation in boreal forest soil
Source: Nat Commun. 2019 Sep 4;10:3982. doi: 10.1038/s41467-019-11993-1 (PMC6726645; doi:10.1038/s41467-019-11993-1)
Supplement: Supplementary file 1 — Supplementary Information [file 41467_2019_11993_MOESM1_ESM.pdf]

Supplementary Information for

Plant roots increase both decomposition and stable soil organic matter formation in boreal forest soil

Adamczyk et al.

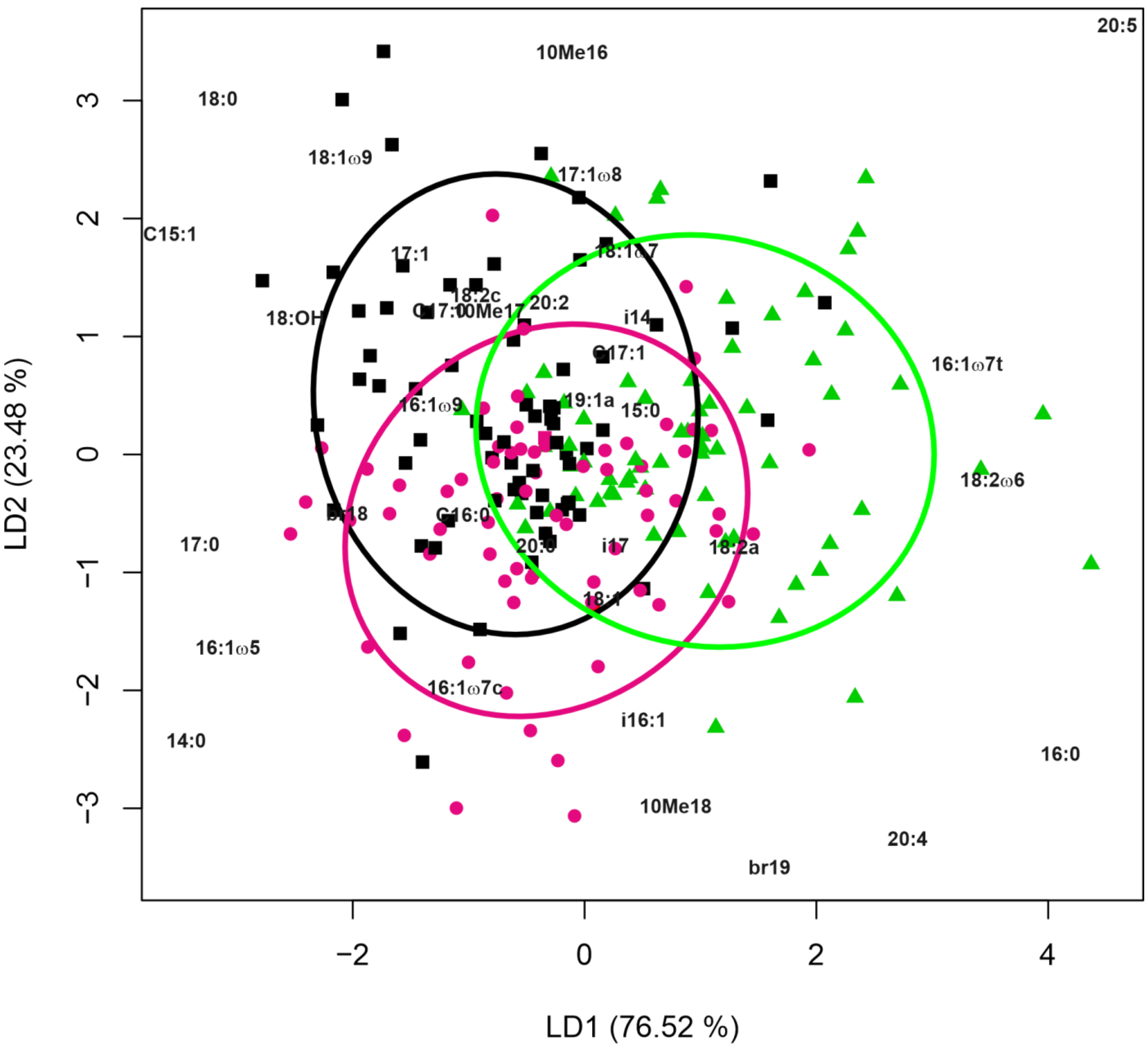

Supplementary Figure 1 Linear discriminant analysis (LDA) illustrating shifts in the microbial community PLFA profile induced by the mesh treatments. The different mesh treatments are represented with green triangles (1000 µm mesh), magenta circles (50 µm mesh) and black squares (1 µm mesh). Source data are provided as a Source Data file.

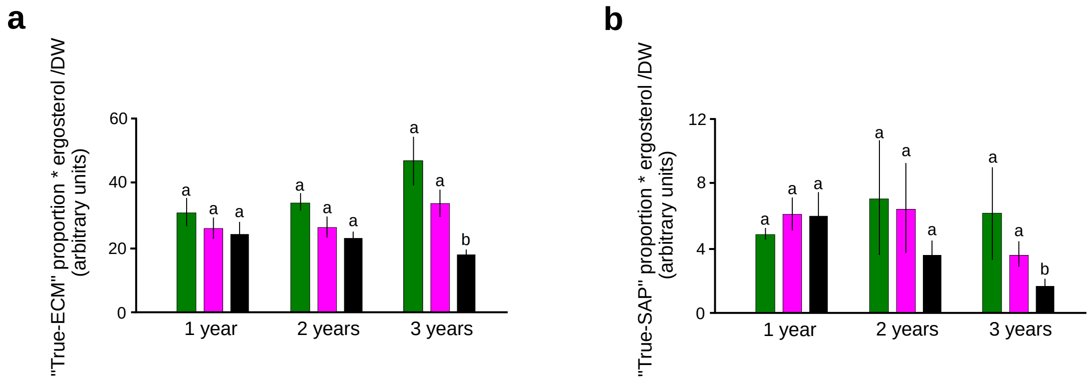

Supplementary Figure 2 Proportion of sequences assigned with FUNGuild[54] to a) ectomycorrhizal (ECM) and b) saprotrophic (SAP) guilds (n=15) relativized with ergosterol µg/DW in different treatments. The sequence data were analyzed by and summarized from Sietiö (2018). The different mesh sizes are represented with different colours (from left to right): 1000 µm (green), 50 µm (magenta) and 1 µm (black). Significant differences (P<0.05, non-parametric Kruskal Wallis test) between treatments within one year are indicated with different letters. The error bars represent ± s.e.m. Source data are provided as a Source Data file.

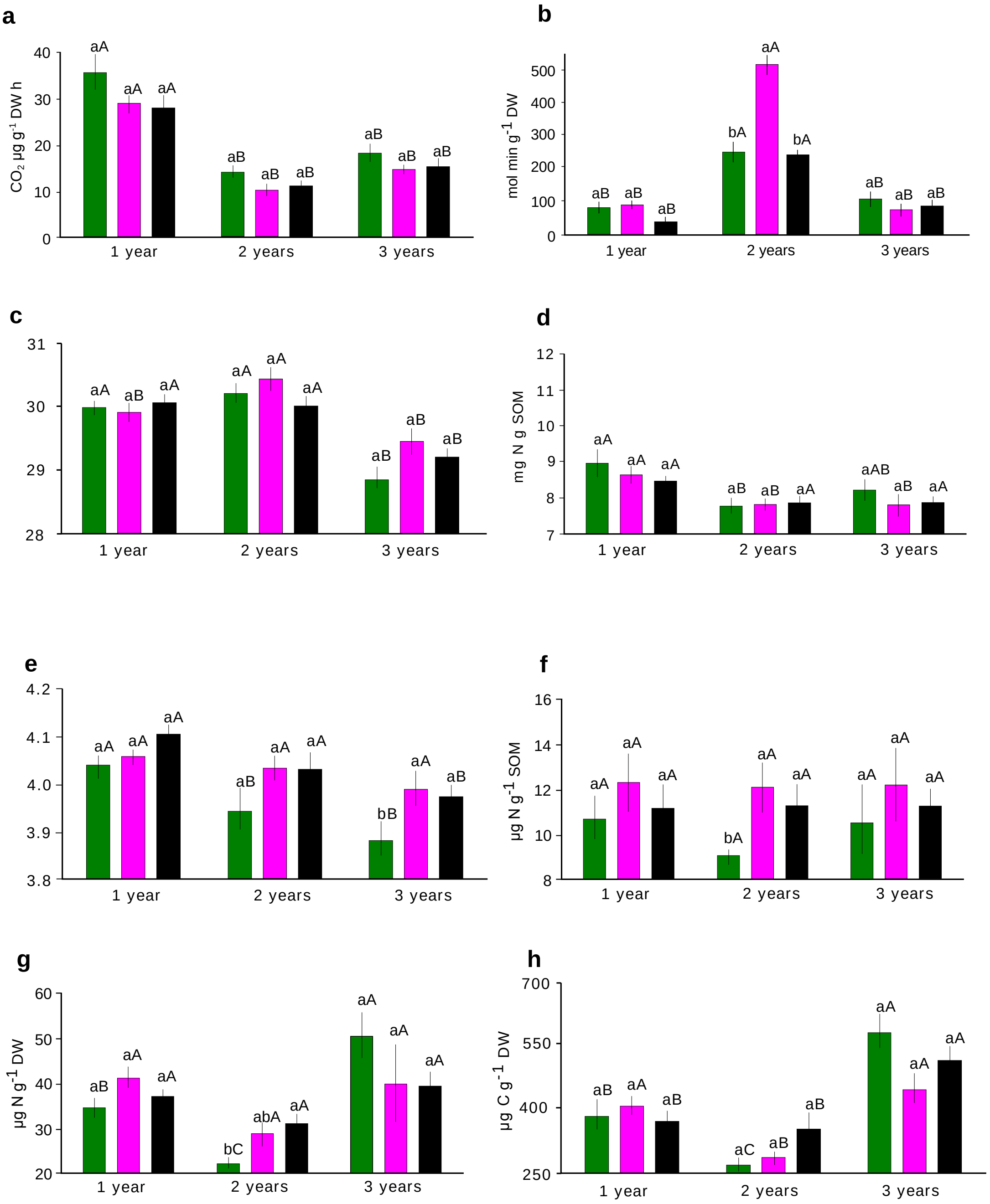

Supplementary Figure 3 Soil chemistry and microbial activities. (**a**) Carbon dioxide (CO_2) production (soil respiration), (**b**) sums of enzymatic activities (for activities of separate enzymes, see Supplementary Fig. 4), (**c**) C-N ratio, (**d**) degradable N pool, (**e**) pH, (**f**) total free amino acids (TFAA), (**g**) total dissolved nitrogen, (**h**) dissolved organic carbon. The different mesh sizes are represented with different colours (from left to right): 1000 µm (green), 50 µm (magenta) and 1 µm (black). The given values are the means of 24 replicates (9 for degradable N pool, TFAA, TDN and DOC). Significant differences ($P<0.05$) between treatments within one year are indicated by different letters, and differences between different years for the same treatment are indicated by capitals. The error bars represent ± s.e.m. Source data are provided as a Source Data file.

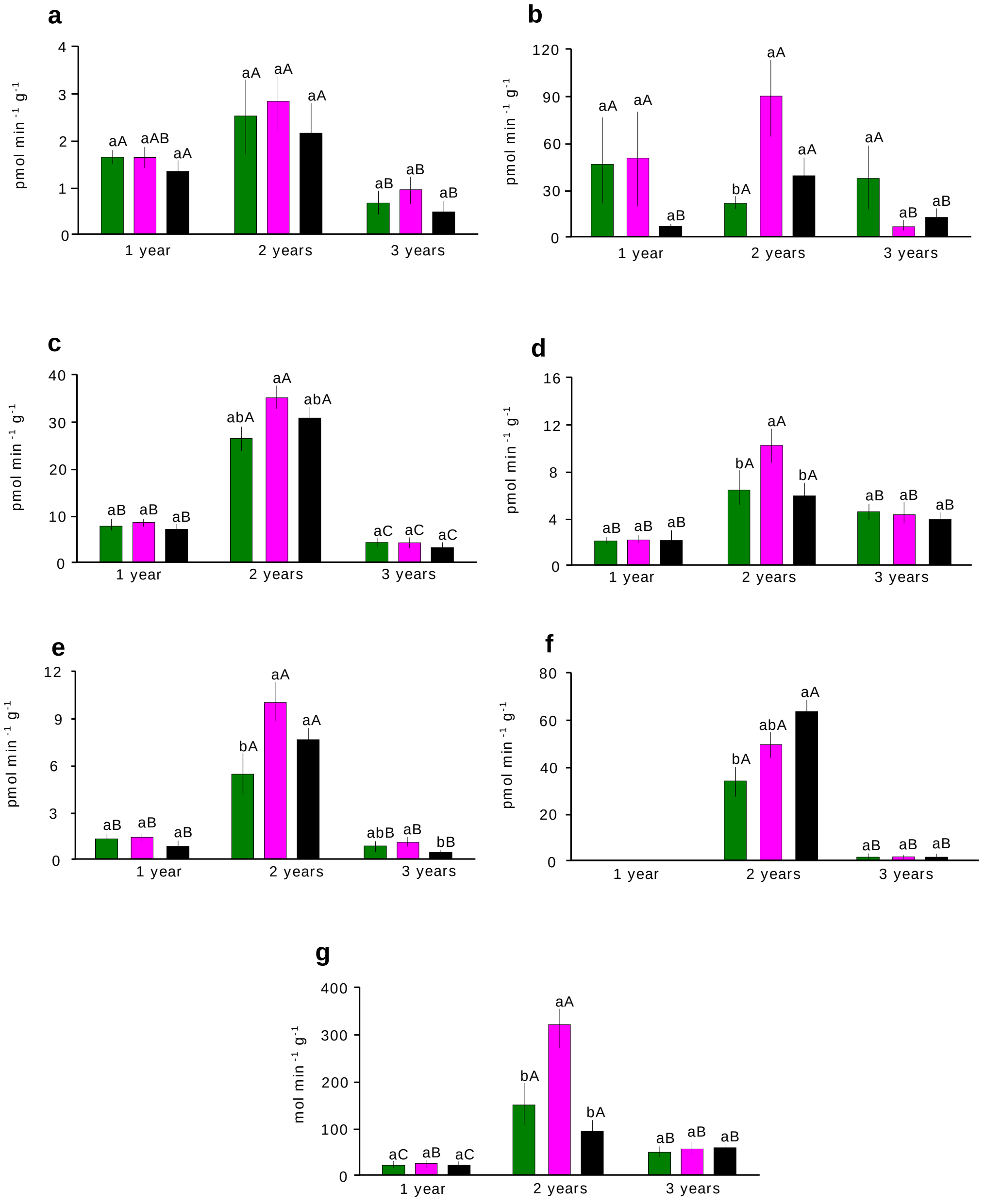

Supplementary Figure 4 Soil enzyme activities: (**a**) cellobiohydrolase I, (**b**) chitinase, (**c**) beta-glucosidase, (**d**) beta-xylosidase, (**e**) beta-glucuronidase, (**f**) leucine amino-peptidase, (**g**) acid phosphatase. The different mesh sizes are represented with different colours (from left to right): 1000 μm (green), 50 μm (magenta) and 1 μm (black). The given values are the means of 24 replicates. Significant differences ($P<0.05$) between treatments within the same year are indicated by different letters, and differences between years for the same treatment are indicated by capitals. The error bars represent ± s.e.m. Source data are provided as a Source Data file.

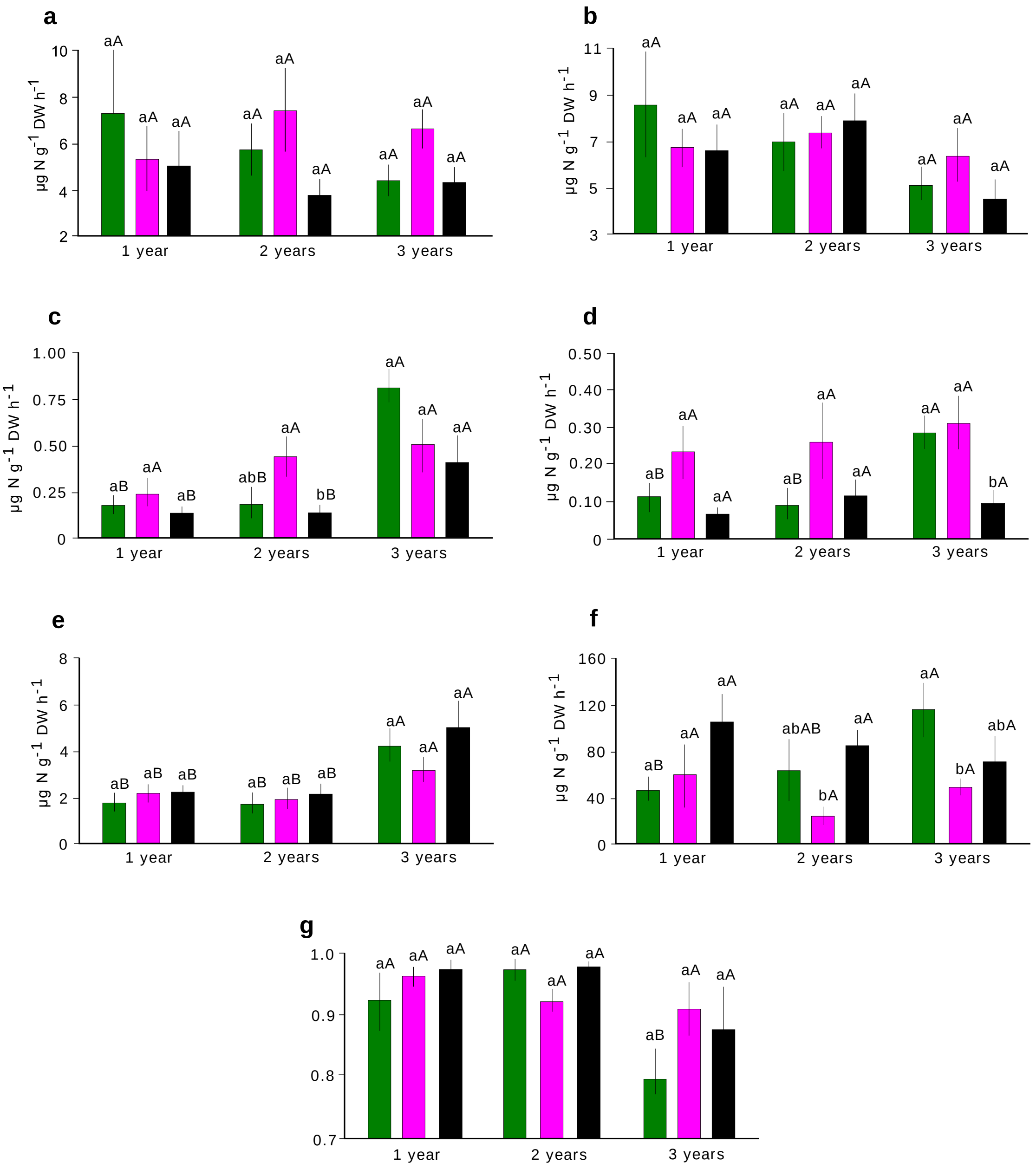

Supplementary Figure 5 Results of ^{15}N pool dilution assays; (**a**) gross protein depolymerization, (**b**) gross amino acid (AA) uptake, (**c**) gross N mineralization, (**d**) gross NH_4^+ uptake, (**e**) total free amino acid (TFAA) mean residence time (MRT), (**f**) NH_4-N mean residence time, (**g**) nitrogen use efficiency (NUE). The different mesh sizes are represented with different colours (from left to right): 1000 µm (green), 50 µm (magenta) and 1 µm (black). The given values are the means of 9 replicates. Significant differences (P<0.05) between treatments within one year are indicated by different letters, and differences between different years for the same treatment are indicated by capitals. The error bars represent ± s.e.m. Source data are provided as a Source Data file.

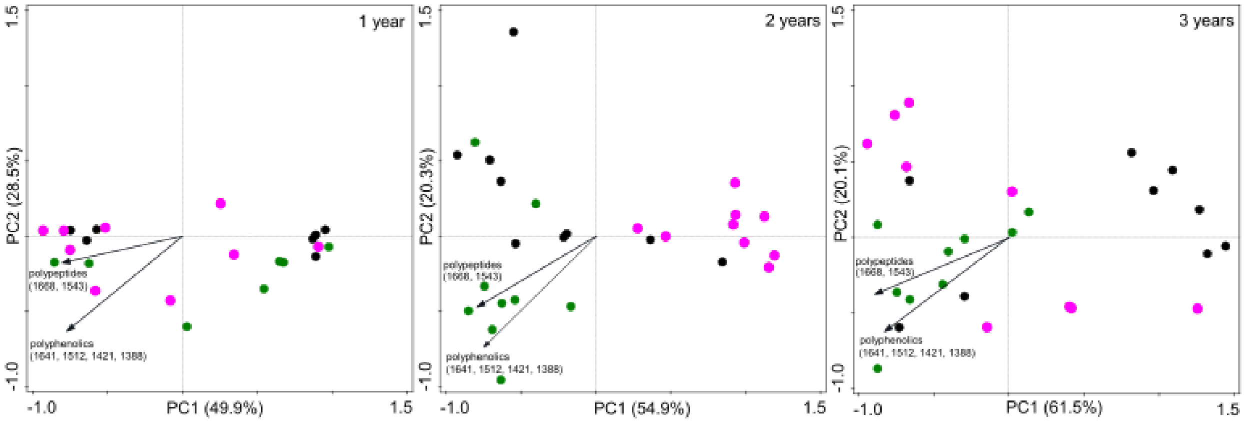

Supplementary Figure 6 Ordination diagram from the PC analysis showing the treatment effect of on FTIR-derived soil chemistry. Arrows represent wavenumbers that were most affected by treatment (showed best fit to PC ordination space). For simplicity, the absorbance values at the wavenumbers assigned to polypeptides (1668 cm^{-1} and 1543 cm^{-1}) or polyphenolics (1641 cm^{-1}, 1512 cm^{-1}, 1421 cm^{-1} and 1388 cm^{-1}) were summed and projected into the existing ordination space as supplementary variables. The different mesh sizes are represented by different colours: 1000 µm (green), 50 µm (magenta) and 1 µm (black). Based on IR spectroscopy, soil chemistry differed between treatments in the second and third years ($F=11.8$, $P<0.005$ for the second year; $F=3.5$, $P<0.01$ for the third year); the effect of the year for a given mesh size was also significant for the second and third years ($F=5.9$, $P<0.005$ for the second year; $F=3.5$, $P<0.005$ for the third year).

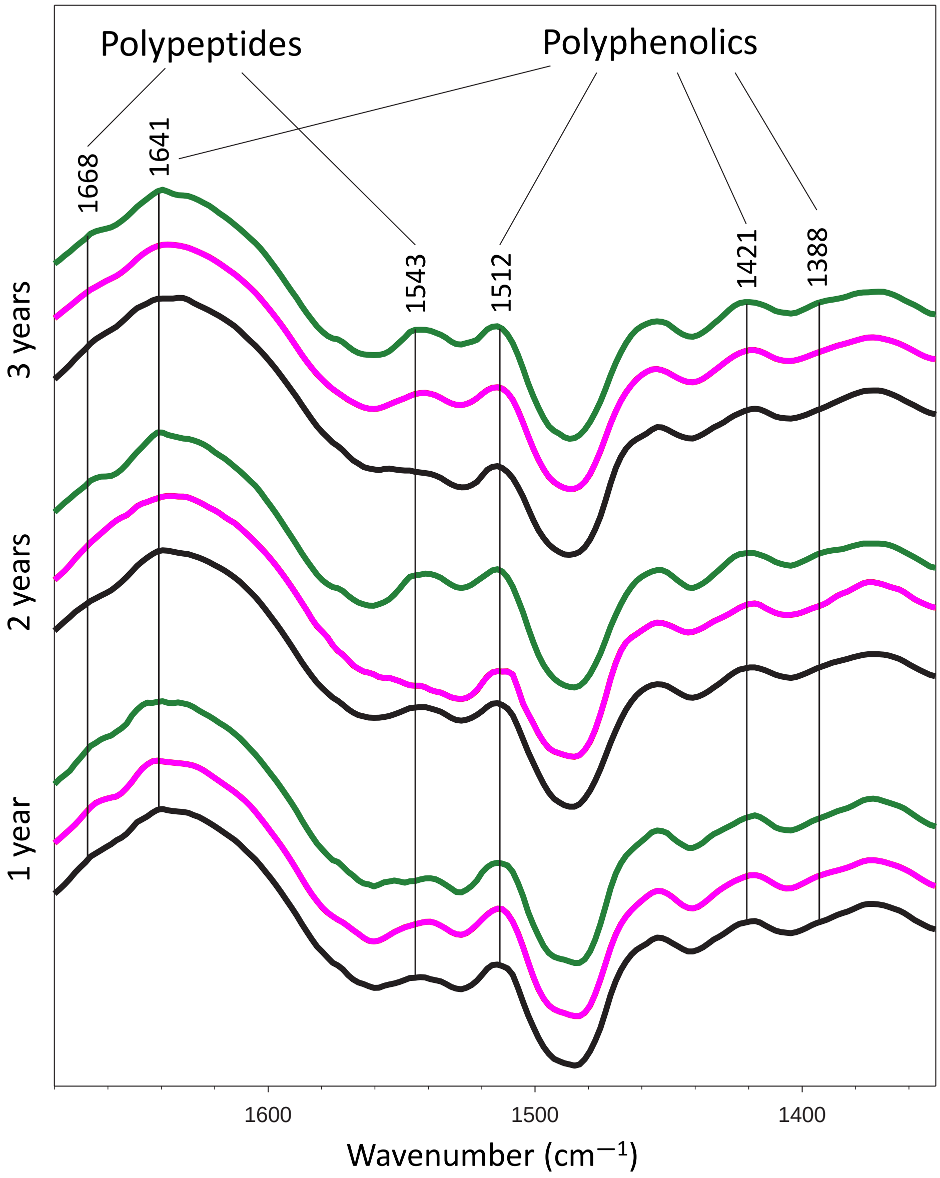

Supplementary Figure 7 FTIR spectra of soils; bands that were most affected by treatment are marked in grey and emphasized in the upper graphs, where the band heights represent the vertical distance from a local baseline. The different mesh sizes are represented with different colours 1000 µm (green), 50 µm (magenta) and 1 µm (black). Bands were assigned based on a previous study[3].

Supplementary Table 1. Root ingrowth, soil moisture and fungal biomass (shown as ergosterol as biomarker). Average values (n=24, for ergosterol n=3, pooled samples) ± s.e.m. Significant differences (P<0.05) between treatments within one year are indicated by different letters, and the differences between different years for the same treatment are indicated by capitals.

year	treatment	root ingrowth (g)	soil moisture (%)	ergosterol (μg/g DW)
1st year	1μm	0.00	64.6(0.49)^{aA}	59.3(2.17)^{abA}
	50μm	0.00	66.1(0.64)^{aA}	65.0(1.97)^{aA}
	1000μm	0.08(0.015)^{A}	64.9(0.57)^{aA}	66.7(1.27)^{aC}
2nd year	1μm	0.00	55.9(1.93)^{aB}	45.4(2.12)^{cB}
	50μm	0.00	56.7(1.93)^{aB}	57.3(3.27)^{bB}
	1000μm	0.24(0.042)^{B}	50.8(1.66)^{bB}	67.5(2.46)^{aB}
3rd year	1μm	0.00	48.4(2.49)^{aB}	32.3(3.11)^{cC}
	50μm	0.00	45.8(2.36)^{aC}	65.8(1.81)^{bA}
	1000μm	0.27(0.035)^{C}	42.7(2.60)^{aC}	80.9(4.73)^{aA}

Supplementary Table 2. Soil micro- and mesofauna, averages (n=3, pooled samples) ± s.e.m. Significant differences ($P<0.05$) between treatments within one year are indicated by different letters, and the differences between different years for the same treatment are indicated by capitals. FW – fresh weight.

**year**	**treatment**	**Nematodes (individual per g soil FW)**	**Oribatid mites (Individual per g soil FW)**	**Other mites (mesostigmata/astigmata per g soil FW)**	**Enchytraeids (individual per g soil FW)**
1st year	1µm	0.36(0.36)^{aB}	39.1(12.50)^{aA}	0.16(0.16)^{aA}	0
	50µm	0.33(0.57)^{aB}	7.43(4.76)^{bB}	0^{aA}	0
	1000µm	0.16(0.28)^{bB}	10.66(1.19)^{bC}	0.53(0.31)^{aA}	0
2nd year	1µm	0.73(0.36)^{aB}	30.36(4.16)^{aA}	0.16(0.16)^{aA}	0
	50µm	1.83(1.04)^{aB}	20.6(0.36)^{bA}	0^{aA}	0
	1000µm	0.73(0.46)^{aB}	35.16(2.57)^{aA}	0.16(0.16)^{aA}	0
3rd year	1µm	2.26(0.05)^{aA}	23.6(1.41)^{aB}	0^{aA}	0a
	50µm	2.46(0.73)^{aA}	16.63(5.93)^{aB}	0^{aA}	0.33(0.16)^{a}
	1000µm	2.73(0.43)^{aA}	18.86(1.96)^{aB}	0.2(0.2)^{aA}	0.2(0.2)^{a}
sieved and homogenized soil before placing into mesh bags		2.10(0.26)	2.71(1.06)	0.1(0.1)	0

Supplementary References

1. Nguyen, N. H. *et al.* FUNGuild: An open annotation tool for parsing fungal community datasets by ecological guild. *Fungal Ecol.* **20,** 241–248 (2016).

2. Sietiö, O.-M. The role of plant-fungal interaction for the soil organic matter degradation in boreal forest ecosystem. (University of Helsinki, 2018).

3. Adamczyk, B. *et al.* The contribution of ericoid plants to soil nitrogen chemistry and and organic matter composition in boreal forest soil. *Soil Biol. Biochem.* **103,** 394–404 (2016).
