# Supplementary material for: Plant roots increase both decomposition and stable organic matter formation in boreal forest soil
Source: Nat Commun. 2019 Sep 4;10:3982. doi: 10.1038/s41467-019-11993-1 (PMC6726645; doi:10.1038/s41467-019-11993-1)
Supplement: Supplementary file 3 — Reporting Summary [file 41467_2019_11993_MOESM3_ESM.pdf]

nature research

Corresponding author(s): Bartosz Adamczyk

Last updated by author(s): 21.7.2019

Reporting Summary

Nature Research wishes to improve the reproducibility of the work that we publish. This form provides structure for consistency and transparency in reporting. For further information on Nature Research policies, see Authors & Referees and the Editorial Policy Checklist.

Statistics

For all statistical analyses, confirm that the following items are present in the figure legend, table legend, main text, or Methods section.

n/a	Confirmed	
☐	☒	The exact sample size (n) for each experimental group/condition, given as a discrete number and unit of measurement
☒	☐	A statement on whether measurements were taken from distinct samples or whether the same sample was measured repeatedly
☐	☒	The statistical test(s) used AND whether they are one- or two-sided<br>*Only common tests should be described solely by name; describe more complex techniques in the Methods section.*
☐	☒	A description of all covariates tested
☐	☒	A description of any assumptions or corrections, such as tests of normality and adjustment for multiple comparisons
☐	☒	A full description of the statistical parameters including central tendency (e.g. means) or other basic estimates (e.g. regression coefficient) AND variation (e.g. standard deviation) or associated estimates of uncertainty (e.g. confidence intervals)
☐	☒	For null hypothesis testing, the test statistic (e.g. F, t, r) with confidence intervals, effect sizes, degrees of freedom and P value noted<br>*Give P values as exact values whenever suitable.*
☒	☐	For Bayesian analysis, information on the choice of priors and Markov chain Monte Carlo settings
☒	☐	For hierarchical and complex designs, identification of the appropriate level for tests and full reporting of outcomes
☐	☒	Estimates of effect sizes (e.g. Cohen's d, Pearson's r), indicating how they were calculated

Our web collection on statistics for biologists contains articles on many of the points above.

Software and code

Policy information about availability of computer code

Data collection	Chemstation software (CO2 and chitin); IRsolution 1.40 software (for FTIR); Magellan software (plate reader)
Data analysis	Chemstation software (CO2 and chitin); IRsolution 1.40 software, Unscrambler, Canoco 5 (for FTIR); Magellan software (plate reader), Isodat (15N); R software and SPSS (statistics)

For manuscripts utilizing custom algorithms or software that are central to the research but not yet described in published literature, software must be made available to editors/reviewers. We strongly encourage code deposition in a community repository (e.g. GitHub). See the Nature Research guidelines for submitting code & software for further information.

Data

Policy information about availability of data

All manuscripts must include a data availability statement. This statement should provide the following information, where applicable:

- Accession codes, unique identifiers, or web links for publicly available datasets
- A list of figures that have associated raw data
- A description of any restrictions on data availability

The data that support the findings of this study are available on request from the corresponding author (B.A.). The source data underlying Figs and Supplementary Figs are provided as a Source Data file.

Field-specific reporting

Please select the one below that is the best fit for your research. If you are not sure, read the appropriate sections before making your selection.

☐ Life sciences ☐ Behavioural & social sciences ☒ Ecological, evolutionary & environmental sciences

For a reference copy of the document with all sections, see nature.com/documents/nr-reporting-summary-flat.pdf

Ecological, evolutionary & environmental sciences study design

All studies must disclose on these points even when the disclosure is negative.

Study description	The soil for this study was collected in the vicinity of the Station for Measuring Forest Ecosystem-Atmosphere Relations (SMEAR II) of Helsinki University in Hyytiälä (61°84'N, 24°26'E) in southern Finland. The soil was a haplic Podzol and Scots pine (Pinus sylvestris L.) was the dominant tree species. The soil was taken from the organic layer (Ofh) and, after the removal of visible roots, homogenized and sieved through a 4 mm mesh. The sieved, unsterilized soil was placed in mesh bags with different mesh sizes to form three types of treatments. Mesh bags in 24 replicates per treatment were placed on the topmost mineral horizon, but within the soil organic layer in May 2013 and collected every September in 2013, 2014 and 2015. Three mesh sizes were compared that excluded the ingrowth of plant roots as well as mycorrhizal hyphae (1 µm mesh size treatment), permitted the ingrowth of hyphae but not of roots (50 µm mesh size treatment) or permitted the ingrowth of both hyphae and roots (1000 µm mesh size treatment). We compared SOM loss and stable SOM-N formation, soil chemistry and fauna, microbial community structure and activities (enzymatic activity and microbial N transformations) between the mesh size treatments, focusing specifically on N pools and fluxes.
Research sample	We used soil samples incubated in mesh bags of different mesh sizes. These soil samples were used to study SOM loss and stable SOM-N formation, soil chemistry and fauna, microbial community structure and activities (enzymatic activity and microbial N transformations).
Sampling strategy	After removal from the soil, the mesh bags were first used to study CO2 production (at +4C), Then, the bottles were opened and transported at +4 °C in the dark to a central laboratory and, after removing the visible roots , samples were weighed and prepared for different measurements (see Supplementary Information for each measurement procedure).
Data collection	Mesh bags were collected every September in 2013, 2014 and 2015. All of the authors were involved in sample collection and/or analysis.
Timing and spatial scale	Samples were taken each September in 2013, 2014, and 2015, so at the end of growing season in Finland. By doing so, we were able to observe the changes in humus soil placed in mesh bags of different mesh sizes (treatments) after 1st, 2nd and 3rd year of incubation at study site.
Data exclusions	On principle, data were only excluded, however, if the mesh bag was visibly broken, it was excluded from analysis.
Reproducibility	To avoid biases associated with having multiple laboratories analyzing soils, most of the analyses were done in the same lab (University of Helsinki) only PLFA analyses were done in Luke (at the same campus as University of Helsinki) and 15N dilution pool was analyzed abroad (University of Vienna).
Randomization	Soil samples were randomly processed for analyses.
Blinding	Blinding was not relevant for this study.

Did the study involve field work? ☒ Yes ☐ No

Field work, collection and transport

Field conditions	The experiment was conducted at The Station for Measuring Forest Ecosystem-Atmosphere Relations (SMEAR II) of Helsinki University in Hyytiälä (61°84'N, 24°26'E) in southern Finland. At this site mean temperature is +3C and mean precipitation is 700 mm.
Location	The Station for Measuring Forest Ecosystem-Atmosphere Relations (SMEAR II) of Helsinki University in Hyytiälä (61°84'N, 24° 26'E) in southern Finland.
Access and import/export	Not applicable for this study.
Disturbance	Not applicable for this study.

Reporting for specific materials, systems and methods

We require information from authors about some types of materials, experimental systems and methods used in many studies. Here, indicate whether each material, system or method listed is relevant to your study. If you are not sure if a list item applies to your research, read the appropriate section before selecting a response.

Materials & experimental systems

n/a	Involved in the study
☒	☐ Antibodies
☒	☐ Eukaryotic cell lines
☒	☐ Palaeontology
☒	☐ Animals and other organisms
☒	☐ Human research participants
☒	☐ Clinical data

Methods

n/a	Involved in the study
☒	☐ ChIP-seq
☒	☐ Flow cytometry
☒	☐ MRI-based neuroimaging
